# Alloys for Filling Purposes

**Published:** 1900-06

**Authors:** A. P. Fellows

**Affiliations:** Philadelphia


### ALLOYS FOR FILLING PURPOSES.[1]

BY A. P. FELLOWS, D.D.S., PHILADELPHIA.

I HAVE brought together in this paper my experiences, ranging over a period of a dozen years, with the furnace and crucible and the alloys when ready for use, and shall only attempt to mention some of the things which modify the working qualities of amalgams.

What more fitting time than this to be truly professional in regard to the materials we use in our practice? We would not use or prescribe a medicine of which we did not know its component parts and its action. Why should we do differently with our alloys? Do we know the ingredients of the alloys we are using daily, and what their qualities are, and why those qualities are present or absent? If we do not, is it not time to begin? Is it not common

---

[1] Read before the Academy of Stomatology, January 23, 1900.

to buy an ounce of "amalgam" and use that for every tooth that requires amalgam, and when that quantity is used to buy another ounce of whatever alloy a friend recommends, not knowing or caring what the formula may be, or how it is made, or what qualities the alloy possesses? I say again, is it not time to begin to be professional, to use only such alloys as are accompanied by printed formulæ?

For many years we have been seeking a material for filling teeth which may be inserted in a plastic condition, requiring no great pressure in insertion, which will not injure the pulp or supporting walls; which will become hard enough after setting to stand the stress of mastication without changing its form, and neither shrink nor expand.

Years ago an amalgam of pure silver and mercury was tried, and at the present time silver is the base of all our dental alloys, varying percentages of other metals being combined with it to give peculiar qualities. The metals in general use besides silver are tin, gold, copper, zinc, platinum, and sometimes antimony, bismuth, cadmium, and other metals, named aproximately in order of usefulness.

Tin added to the silver gives an alloy which amalgamates more readily and thoroughly than pure silver, and the amalgam sets more slowly, and is more stable and generally more workable. Gold adds edge-strength; also increases the speed of setting. Copper adds edge-strength, but reduces the speed of setting. Zinc reduces edge-strength and speed of setting, but also reduces the tendency to contract. Platinum is supposed to give hardness to an alloy, but it is of questionable utility. All the other metals have been discarded.

I have noticed that in proportion to the increase in the percentage of silver, there is an increase in the amount of mercury required, an increase in the difficulty of amalgamation, an increase in the hardness of the amalgam up to certain limits, and an increase in the speed of setting and also in the tendency to bulge or expand, and conversely as the percentage of silver is decreased there is an increase in the property of easy and thorough amalgamation, the alloy rubbing up more smoothly with the mercury either in the palm or in the mortar. We also find the amalgam slower setting and softer when set. The filling will not stand the strain of mastication as well, and will have less edge-strength. The use of silver in our dental alloys varies from forty to seventy per cent.

Tin stands second in usefulness for dental alloys, and varies in its proportions from twenty-five to sixty per cent. Undoubtedly its greatest use is to facilitate amalgamation. It has therapeutic properties; it renders the filling less conducting, also softer, and gives a better and more stable color; but since it oxidizes readily, and the alloy in which it enters to any great extent is soft, its action should be controlled, and so we look for a metal which will counterbalance its action, which is gold.

Gold, therefore, takes third place in dental alloys, and ranges in proportion from a fraction of one per cent. to ten per cent. Gold gives certain properties peculiar to itself. Alone it has a great affinity for mercury, but as soon as it is alloyed with other metals it loses that affinity to some extent. I have not noticed a greater ease in amalgamation of an alloy containing gold than one not containing it. Indeed, it has always appeared to me, though I may be mistaken, that an alloy containing gold does not amalgamate as readily as the same where gold was absent. It does give more hardness to an alloy than an equal precentage of any other metal.

Gold controls color, in that it is not acted upon by any acid or by the atmosphere. It gives an excellent edge-strength, and also renders the alloy quick-setting, which at times may be put to good service, but I think that in general practice too rapidly setting alloys are not advisable, as there is a greater chance of shrinkage, and one does not have time for the proper packing which is so absolutely necessary in the making of a correct filling. No doubt all of us have seen fillings which did not conform to the inside surface of the cavity properly, though they appeared to be good from the outside. I think this is due in part to the use of too rapid setting alloys, also to use of too large pieces, etc.

Copper imparts much the same qualities that gold does, and it is a question whether it should not rank ahead of gold in the making of alloys. No metal we use is more stable, neither shrinking nor expanding. It permits easy and thorough amalgamation, and causes slowness of setting, thereby controlling the metals which produce more rapid setting. It imparts good edge-strength, no fillings retaining better edges than those containing a large percentage of copper. When in combination with gold, the tendency to tarnish is so reduced that one can not detect the presence of copper by the color. I have fillings in which copper is present in good percentage, and in years of use there has been no tooth discoloration and but slight tarnish to the filling. I would venture to say that there are few alloys used in every-day practice which do not contain copper to a certain extent, though we may not be aware of it. We know that there is an alloy of copper, zinc, and platinum which will withstand the action of nitric acid, and no doubt gold gives this same property to an alloy containing copper.

Alloys containing zinc amalgamate quite readily, but not as smoothly as those largely tin. To the amalgam it gives whiteness, slowness of setting, and it controls shrinkage. An alloy containing zinc will not reach its full degree of hardness for a considerably greater time than the same alloy minus the zinc.

The other metals mentioned have been pretty generally discarded in the making of dental alloys, as their bad qualities overbalance the good ones. We cannot be too careful in the choice and handling of an alloy for filling, to get the advantage of useful qualities. One cannot amalgamate an alloy in the palm of the hand properly; if the alloy does amalgamate without pressure and grinding, it will probably make a soft amalgam. We are too apt to think "it is only an amalgam filling." Should we not use just as great pains with a filling of amalgam as with one of gold, since there are so many elements which vitiate the usefulness of the filling?

We should use the rubber dam, prepare the cavity, and insert the amalgam properly, after correctly choosing and mixing the alloy, and last, though not least, finish the filling as carefully as we would a gold filling. Other things being equal, I believe the filling will have no superior in so far as service and durability are concerned.

I have noticed that there are various things which will have a marked effect on the working of an alloy and the stability of the amalgam filling when finished. Chief among these are aging, oxidation, and quantity of mercury used, as well as the mode of mixing.

An alloy may be mixed with less mercury and more thoroughly in the mortar than in the palm, the resulting amalgam being harder when set and withstanding greater stress. In proportion to the decrease in the quantity of mercury the amalgam becomes harder, so long as thorough amalgamation has taken place. I have also noticed that aging, or perhaps surface oxidation, renders the alloy more workable with less mercury, and that annealing while air is excluded will also give the same result with the addition of a less tendency to shrinkage.

Fresh cut alloys require too much mercury to get the degree of hardness which they should have, and they set altogether too rapidly.

In my experience in making alloys I have noticed that there is a great tendency on the part of nearly all the metals used to oxidize under the degree of heat necessary to thoroughly melt them; if the heat is not sufficiently intense the probabilities are that a portion of the silver or gold will remain in the bottom of the crucible, necessitating a remelt, and indicating that the ingot is not homogeneous.

To overcome this difficulty I have resorted to various devices, with varying success. I have covered the surface of the melts with various substances, to protect them from the air while being heated, in order to prevent oxidation so that the heat could be raised to a temperature sufficient to thoroughly melt all the metals and thoroughly mix them. I have also granulated the melt and remelted; also pulverized the melt and sifted and stirred till the mass of powder was thoroughly mixed, and then remelted, bringing the heat just high enough to melt, and then quickly casting in an ingot mould which was cold, in order that the melt might be quickly chilled and prevent the heavier metals from sinking to the bottom. This latter way has succeeded in giving a homogeneous alloy, and one with a loss of but six grains in ten ounces, part of which was accounted for.

If one will take a piece of iron, bring it to a white heat, and dip it into flowers of sulphur, it will at once melt, though the temperature will be considerably below the melting-point, showing the affinity that heated iron has for sulphur. The quality of the iron will be totally destroyed.

Would it not be reasonable to believe that other metals in a molten condition would have an affinity for the sulphur thrown off from the fuel in process of combustion, and that their qualities would be impaired in proportion to the amount of the vapors of sulphur present and taken up just as in the case of iron? May not other gases be present in the furnace which act readily on the melted metals to their detriment?

After an ingot has been made it is necessary to reduce it to very small particles, in order that the mercury can readily act upon it. This has been done by filing or turning, the one giving fine particles, the other thin flakes. We know an alloy will be taken up by mercury in proportion to the surface exposed; therefore the finer the powder the more readily it is amalgamated. If these sheets of turned or planed alloy were pulverized, or the ingot filed with a very fine file, and thus comminuted to dust-like fineness, I believe we could amalgamate it with more ease and with less mercury, producing therefore a harder amalgam.

